# A Systematic Literature Review of Peer-led Strategies for Promoting Physical Activity Levels of Adolescents

**DOI:** 10.1177/10901981211044988

**Published:** 2021-10-11

**Authors:** Fiona McHale, Kwok Ng, Sarah Taylor, Enrique Bengoechea, Catherine Norton, Donal O’Shea, Catherine Woods

**Affiliations:** 1 University of Limerick, Limerick, Ireland; 2 University of Eastern Finland, Joensuu, Finland; 3 Leeds Trinity University, Leeds, UK; 4 St. Vincent’s University Hospital, Dublin, Ireland

**Keywords:** school-based, peer training, behavior change techniques, social support, demonstration of behavior, information about health consequences

## Abstract

*Background*. Low levels of physical activity (PA) in adolescents highlight the necessity for effective intervention. During adolescence, peer relationships can be a fundamental aspect of adopting and maintaining positive health behaviors. *Aim*. This review aims to determine peer-led strategies that showed promise to improve PA levels of adolescents. It will also identify patterns across these interventions, including training provided and the behavior change techniques (BCTs) employed. *Method*. Adhering to Preferred Reporting Items for Systematic Reviews and Meta-Analyses guidelines, PubMed, PsychINFO, and Scopus were searched using key concepts of peer, PA and adolescent for articles that examined interventions that had a peer-led component and reported on at least one PA outcome in 12- to 19-year-olds. Following title and abstract screening of 1,509 studies, and full text review stage, 18 progressed to data synthesis. Methodological quality was assessed using an adapted scale. *Results*. Quality assessment identified 11 studies as high quality. Half of the included studies (*n* = 9) reported improved PA outcomes in the school setting. The most prominent behavioral change techniques were social support, information about health consequences, and demonstration of the behavior. Older adolescents leading younger peers and younger adolescents leading those of the same age showed potential. Seldom have older adolescents been targeted. Gender-specific interventions showed the most promise. *Conclusion*. Peer leadership requires careful planning and in the school setting can be a resourceful way of promoting adolescent PA.

## Background

Despite the physical, social, mental, and cognitive health benefits favorably associated with physical activity (PA), 84.7% of girls and 77.6% of boys globally were identified as insufficiently active to meet recommended daily PA guidelines of 60 minutes of moderate-to-vigorous-physical-activity (MVPA; Guthold et al., 2020). Although the guidelines have now been updated, no longer requiring daily achievement of 60 minutes of MVPA, the level of PA decline throughout adolescence remain a global concern (Bull et al., 2020). Additionally, studies consistently show boys to be more active than girls (Currie et al., 2009; Woods et al., 2018) and these habits developed in childhood may track into adulthood (Hardie Murphy et al., 2016; Hayes et al., 2019). Therefore, establishing effective ways to promote more physically active behaviors in the adolescent population are needed.

Adolescence is a critical period in life for health and well-being (Patton et al., 2016). During adolescence, there are physical, social, emotional, and cognitive changes that may lead to new health behaviors (Sawyer et al., 2018) and transitions in family, peer, and educational domains become apparent (Viner et al., 2012). Adolescents are still in a period where they are dependent on adults for protection (Sawyer et al., 2018). Despite this, adolescents begin to distance themselves from parents, have a continued drive for independence, and popularity among friends becomes important (Sawyer et al., 2012). Positive and supportive peers can play a fundamental role in helping young people adopt and maintain positive health behaviors (Mendonça et al., 2014; Salvy et al., 2009) through mechanisms such as social support, companionship, friendship quality, acceptance, and peer crowd affiliation (Fitzgerald et al., 2012). There is evidence for an increase in PA through normalizing PA among peer networks and encouragement for adolescents to be physically active together (Prochnow et al., 2020).

Peer-leadership, peer-mentorship, peer-tutoring, peer-delivered, and peer-assisted learning (PAL) are interchangeable terms frequently used in health and educational literature (Colvin, 2007; Hulteen et al., 2019; Jenkinson et al., 2013). These terms describe any learning process whereby adolescents learn from and with others of similar ages, similar experiences, or those who are older but within the same environment (Colvin, 2007; Jenkinson et al., 2013). For this review, the term peer-led will be all encompassing to describe interacting with and motivating peers to initiate, continue, and sustain positive behavior (Barr-Anderson et al., 2012; Campbell et al., 2008).

School-based interventions deploying peer-led strategies have been widely used for promoting positive health behaviors in adolescents (Sun et al., 2018; Thomas et al., 2015; Yip et al., 2016). There were significant improvements in smoking behaviors (Sun et al., 2018) and positive outcomes for nutrition were reported for knowledge, self-efficacy, attitudes toward healthy eating, dietary measures, and physical health measures (Yip et al., 2016). Additionally, for sexual health interventions, knowledge, and attitudes were significantly improved, however, behavior change outcomes were not statistically significant (Sun et al., 2018). Four reviews have examined the effect of peer-led strategies that targeted PA as an outcome measure (Christensen et al., 2020; Ginis et al., 2013; Hulteen et al., 2019; Jenkinson et al., 2013). One review focused on school-based interventions (Jenkinson et al., 2013), two focused on all age populations (Ginis et al., 2013; Hulteen et al., 2019) and one focused on youth in all settings, that is, school, home, clinical, community, and university (Christensen et al., 2020). One review found evidence for increased PA, however, only reported on five studies for the adolescent population (Hulteen et al., 2019). A scoping review found evidence for improved PA outcomes among youth peer leaders along with increased knowledge and attitudes toward health, enhanced confidence, enjoyment of being a role model, and development of leadership skills in peer leaders (Christensen et al., 2020). A review by Jenkinson et al. (2013) reported just two studies focused on adolescents with PA as an outcome measure (Lubans et al., 2011; Peralta et al., 2009). These previous reviews pointed to the need for further research to clarify (1) patterns associated with impact, that is, peer-leaders characteristics, intervention components, or underlying theory (Christensen et al., 2020); (2) the training requirements for peer leaders; (3) the most promising behavior change techniques (BCTs) to elicit behavior change (Hulteen et al., 2019) and identification of the ideal strategies to promote peer-leadership programs with particular focus on adolescents (Jenkinson et al., 2013).

Consequently, the aim of this review is to provide intervention developers with valuable information about the “active ingredients” that can be targeted in training for peer-leaders using the behavior change technique taxonomy v1 (BCTTv1) (Michie et al., 2013). A BCT is defined as “an observable, replicable, and irreducible component of an intervention designed to alter or redirect causal processes that regulate behavior, that is, a technique is proposed to be an active ingredient” (Michie et al., 2013, p. 82). PA, dietary, and obesity interventions for children and adolescents have identified and utilized BCTs in their intervention development, for example, social support (practical) and goal setting (Brannon & Cushing, 2015; Hendrie et al., 2012). Although peer-led interventions have potential for promoting positive health behaviors among adolescents (Christensen et al., 2020; Ginis et al., 2013; Hulteen et al., 2019; Jenkinson et al., 2013), inadequate description of interventions often makes identification of effective components difficult (Duff et al., 2017). Therefore, additional research is needed to understand the impact of these interventions and their active components for the enhancement of peer-leadership for health promotion. Further research is required to focus on peer-leadership in the adolescent population and ascertain the training factors for peer leaders and strategies used in the peer-leadership approach with best impact.

To the authors’ knowledge, this is the first systematic review that focuses solely on adolescents in peer-led PA interventions. The primary aim is to identify peer-led programs that showed promise in improving PA levels of 12- to 19-year-old participants and/or peer leaders. The secondary aims are to determine peer-leadership training factors and identify the most promising BCTs employed by peer-led interventions reporting on improved PA outcomes.

## Method

A protocol for this systematic review was prepared and registered with PROSPERO (Registration number: CRD42018090400). The review adhered to the PRISMA (Preferred Reporting Items for Systematic Reviews and Meta-Analyses) reporting guidelines for systematic reviews (Moher et al., 2009).

A comprehensive literature search strategy was used involving both primary and secondary strategies (Shea et al., 2017). The primary strategy involved three electronic databases (PubMed, PsychINFO, and Scopus). Boolean operators were used to expand, exclude, or join keywords in the search, for example (peer* OR mentor* OR leader*) AND (“physical* active*” OR exercise OR “physical educat*” OR sport OR fitness) AND (adolescen* OR teenag* OR youth OR “young adult*” OR pupil* student*). The search, conducted in March 2020 was not restricted by year of publication and was limited to title and abstract only in each database. The secondary search strategy involved reviewing bibliographies from included articles to identify additional relevant studies.

### Eligibility Criteria and Evidence Acquisition

The PICOS (participants, interventions, comparisons, outcomes and study design) framework (Schardt et al., 2007) was used to frame the research questions and the inclusion criteria through the search strategy process. Original studies, peer reviewed and in English language were included. Studies were eligible if all of the following criteria were fulfilled: (1) they reported on the delivery of a PA intervention that was either peer-led or had a peer-led component, (2) they reported on interventions in which both the peer leaders and participants were aged 12 to 19 years, (3) the comparison group were adolescents not exposed to a peer-led intervention, (4) PA was an outcome measure of the study, and (5) PA outcomes were measured by means of self-report or by a device-based measure.

All search results were exported into a reference manager (Endnote X7) and duplicates were removed. Initially the first author (a) screened all titles and abstracts and a random sample (10%) was also checked by another member of the research team (b). The full-text of eligible studies were then retrieved and reviewed by the first author. Two members of the research team (b, c) also checked the full text of all eligible studies. Where there was a disagreement, all three authors discussed to agree on a consensus.

### Data Extraction and Synthesis

The following information were extracted for the narrative synthesis; study design, study duration, whether the intervention was fully led by peers or not, peer component description, theoretical framework underpinning the study, BCTs identified, PA outcomes, PA measurement, method of choosing peer leaders, and the age dynamic between leaders and peers. A breakdown of peer-leadership training to include the length, content, location, follow-up, and training facilitator was extracted separately. Those studies that explicitly reported the use of BCTs were extracted and studies not explicitly reporting the use of BCTs were analyzed and coded for BCTs by the lead author using the BCTTv1 (Michie et al., 2015). A member of the research team (c) completed this task also with any disagreements consulted with a third reviewer (b) for consensus.

### Methodological Quality Assessment

Study quality was assessed using a 10-item scale used in previous work, and was further adapted for the needs of this study (van Sluijs et al., 2007). The scale focused on internal validity and analyses assessing for randomization procedure, participant follow up, a validated measure of PA used, participation drop out, blinding of assessors, unit of analysis, timing of measurements, potential confounders, intention to treat, and comparability of groups at baseline. The scale assessed for each study whether its score on an item was “positive,” “negative,” or “not, or insufficiently, described.” Two reviewers (d, g) independently assessed for one randomly selected study each and together with the first author discussed any disagreement to reach a consensus. Positive scores were accumulated and a study was defined as high quality when it scored ≥6 on a 10-point scale or equivalent where some items were deemed not applicable to a particular study.

## Results

### Literature Search

**Figure 1.**
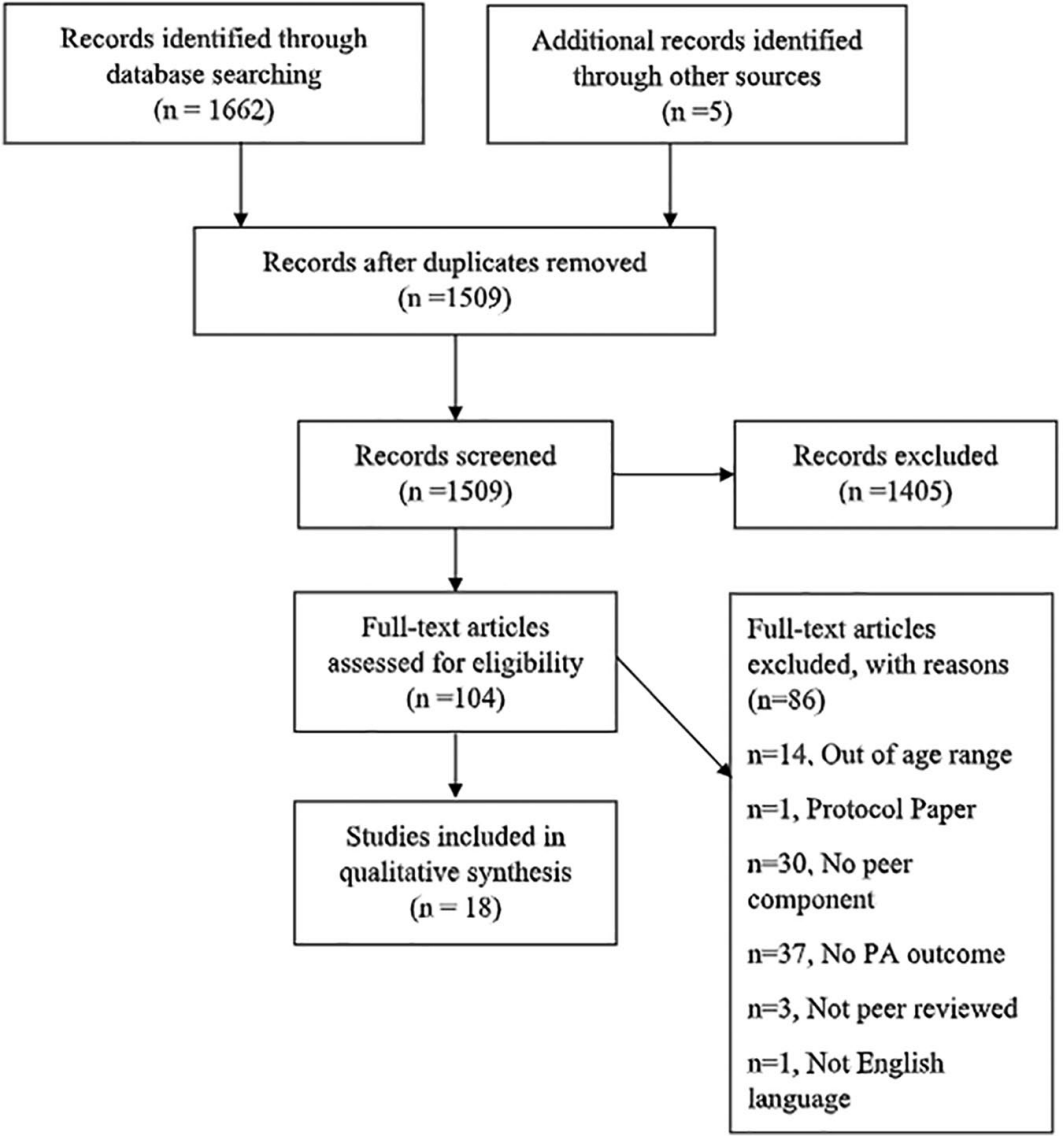
PRISMA flow diagram (Moher et al., 2009) to show each stage of the systematic eligibility process. *Note*. PRISMA = Preferred Reporting Items for Systematic Reviews and Meta-Analyses.

In total, 1,509 publications were identified after duplicates were removed. After screening, 104 studies were assessed for eligibility. Excluded studies were due to participants being out of the age range (*n* = 14), the study had no peer component (*n* = 30) no PA outcome measures were reported (*n* = 37), studies were not peer reviewed (n=3), were a protocol paper (*n* = 1) or were not English language (n=1). 18 studies met the inclusion criteria for the narrative synthesis (Figure 1).

### Study Characteristics

There were seven randomized controlled trials (RCTs; Aceves-Martins et al., 2017; Carlin et al., 2018; Lubans et al., 2011; Lubans et al., 2012; Lubans et al., 2016; Smith et al., 2014; Tymms et al., 2016), including six cluster RCTs (Aceves-Martins et al., 2017; Carlin et al., 2018; Lubans et al., 2012; Lubans et al., 2016; Smith et al., 2014; Tymms et al., 2016); four quasi-experimental studies (Haapala et al., 2017; Jenkinson et al., 2018; Lubans & Morgan, 2008; Utter et al., 2011); one pre–post design (Foley et al., 2017); one exploratory study (Bell et al., 2017); and one crossover study design (Gobbi et al., 2018). There were three feasibility studies (Corder et al., 2016; Owen et al., 2018; Sebire et al., 2018) including a combined feasibility and pilot trial (Corder et al., 2016); and one pilot trial (Cui et al., 2012).

Duration of studies varied from 4 (Cui et al., 2012), 7 (Owen et al., 2018), 8 (Corder et al., 2016; Jenkinson et al., 2018; Lubans & Morgan, 2008), and 12 weeks (Carlin et al., 2018) to 10 (Bell et al., 2017; Corder et al., 2016; Jenkinson et al., 2018) or 12 months (Aceves-Martins et al., 2017; Lubans et al., 2012) and in one instance 3 years (Utter et al., 2011). One study did not report the exact length of the intervention but took place across four class periods (Foley et al., 2017). Eleven studies were assessed as high quality, seven were medium quality and no studies were considered to be low quality (see Supplemental File 1).

### Outcome Measures and Results

Nine studies reported improved levels of PA (Aceves-Martins et al., 2017; Carlin et al., 2018; Corder et al., 2016; Foley et al., 2017; Gobbi et al., 2018; Lubans et al., 2016; Lubans & Morgan, 2008; Owen et al., 2018; Sebire et al., 2018). One study focused only on peer leaders’ outcomes reporting a significant positive effect on PA for peer leader boys (Foley et al., 2017). Five interventions reported statistically significant increases in MVPA (Aceves-Martins et al., 2017; Corder et al., 2016; Foley et al., 2017; Owen et al., 2018; Sebire et al., 2018), two interventions led to statistically significant increases in light intensity PA (LIPA) (Carlin et al., 2018; Gobbi et al., 2018), and two interventions reported statistically significant increases in step count (Lubans et al., 2016; Lubans & Morgan, 2008). Although LIPA and step count increases do not contribute to the MVPA guidelines, increases in step count and LIPA is relevant and important as it fits with the newest PA WHO PA guidelines of “every move counts” (Bull et al., 2020). Nine studies did not report any improved outcomes for PA (Bell et al., 2017; Cui et al., 2012; Haapala et al., 2017; Jenkinson et al., 2018; Lubans et al., 2011; Lubans et al., 2012; Lubans & Morgan, 2008; Smith et al., 2014; Tymms et al., 2016; Utter et al., 2011). PA was measured using accelerometers (Bell et al., 2017; Carlin et al., 2018; Corder et al., 2016; Gobbi et al., 2018; Haapala et al., 2017; Lubans et al., 2012; Lubans et al., 2016; Owen et al., 2018; Sebire et al., 2018; Smith et al., 2014; Tymms et al., 2016), self-reported questionnaires (Aceves-Martins et al., 2017; Cui et al., 2012; Foley et al., 2017; Jenkinson et al., 2018; Utter et al., 2011), and pedometers (Lubans et al., 2011; Lubans & Morgan, 2008). Other positive outcomes included were increased social connectedness, increased school connectedness, increased social self-efficacy (Jenkinson et al., 2018), improved anthropometric measures (Lubans et al., 2011), reduced screen time (Lubans et al., 2012; Smith et al., 2014), improved skill competency (Lubans et al., 2016; Smith et al., 2014), increased motivation (Lubans et al., 2016) increased self-efficacy for PA, increased peer social support, improved well-being, increased PA enjoyment (Corder et al., 2016; Gobbi et al., 2018; Owen et al., 2018; Tymms et al., 2016), and improved muscular fitness (Smith et al., 2014).

### Intervention Characteristics

Only intervention characteristics of the nine studies with improved PA outcomes are reported (detailed information on all included studies can be found in Supplemental File 2). Of the nine studies with improved PA outcomes, seven were fully peer-led (Aceves-Martins et al., 2017; Carlin et al., 2018; Corder et al., 2016; Foley et al., 2017; Gobbi et al., 2018; Owen et al., 2018; Sebire et al., 2018) and two had a peer-led component in the intervention (Lubans et al., 2016; Lubans & Morgan, 2008). All interventions took place in the school setting. See table 1 for summary of intervention characteristics.

**Table 1.** Summary Extraction Table.

	Aceves-Martins et al. (2017)	Carlin et al. (2018)	Corder et al. (2016)	Foley et al (2017)	Gobbi et al. (2017)	Lubans et al. (2008)	Lubans et al. (2016)	Owen et al. (2018)	Sebire et al. (2018)	*n*
Statistically significant PA improvements										
	✓	✓***	✓	✓*	✓***	✓**	✓**	✓		
Intervention type										
Full peer-led intervention	✓	✓	✓	✓	✓			✓	✓	7
Peer-led component						✓	✓			2
Theory										
Social cognitive theory		✓	✓	✓		✓	✓	✓		6
Self-determination							✓	✓		2
Diffusion of innovations theory									✓	1
Youth empowerment/ empowerment educational (behavior) approach	✓			✓						2
Social cognitive theory and self-determination theory							✓	✓		2
Duration										
Intervention <1 academic year		✓	✓			✓	✓	✓		5
Intervention = 1 academic year					✓				✓	2
Intervention >1 academic year	✓									1
Peer dynamic										
Reported training for peer leaders	✓	✓	✓	✓	✓		✓	✓	✓	8
Same age peer leaders	✓				✓	✓	✓		✓	5
Cross aged peer leaders		✓	✓	✓				✓		4
Peer nominated									✓	1
Peer volunteered				✓	✓					2
Peer leader chosen by school	✓		✓					✓		3

*Note*. ✓ Symbol denotes the significant improvements in MVPA in either peers or peer leaders. MVPA = moderate-to-vigorous-physical-activity; LIPA = light intensity PA.

* Only significant MVPA outcomes reported for boys. **Significant improvements in step count. ***Significant improvements in LIPA.

Peer-leadership approaches in studies included presenting information about the intervention to peers and inviting activity suggestions from students (Aceves-Martins et al., 2017), peer-peer informal diffusion of messages about PA (Sebire et al., 2018), a tiered leadership system using older peers and in class peer leaders encouraging participation in PA through activities and a reward system (Corder et al., 2016), delivery of lessons on PA by peer leaders during relevant curriculum time (Foley et al., 2017), buddy systems with typically developing peer leaders buddying up with students with mild–moderate intellectual disabilities for physical education class (Gobbi et al., 2018), modelling and social support through being active with friends (Lubans & Morgan, 2008; Owen et al., 2018) and lunchtime PA sessions run by students (Carlin et al., 2018; Lubans et al., 2016).

### Age Dynamic Between Peer Leaders and Peers

Same aged peer leaders were used in five studies (Aceves-Martins et al., 2017; Gobbi et al., 2018; Lubans et al., 2016; Lubans & Morgan, 2008; Sebire et al., 2018) and four studies used cross aged peer leadership where the peer leader was older in all of the studies (Carlin et al., 2018; Corder et al., 2016; Foley et al., 2017; Owen et al., 2018). With the exception of one study (Jenkinson et al., 2018), in all studies where peer leaders were in mid-late adolescence, that is, 14- to 19-year-olds (Aceves-Martins et al., 2017; Carlin et al., 2018; Corder et al., 2016; Foley et al., 2017; Gobbi et al., 2018; Lubans & Morgan, 2008; Owen et al., 2018) improved PA outcomes were reported. Additionally, with the exception of the study by Jenkinson et al. (2018), all of the studies that did not report improved outcomes comprised peer leaders in early adolescence, that is, 12 to 14 years.

### Method of Choosing Peer Leaders

Teachers picked the peer leaders in three studies (Aceves-Martins et al., 2017; Corder et al., 2016; Owen et al., 2018), peer leaders volunteered in two studies (Foley et al., 2017; Gobbi et al., 2018) and leaders were peer nominated in one study (Sebire et al., 2018). Peer leader choice method was not reported in three studies (Carlin et al., 2018; Lubans et al., 2016; Lubans & Morgan, 2008).

### Theoretical Frameworks

Four studies used social cognitive theory (SCT) (Carlin et al., 2018; Corder et al., 2016; Foley et al., 2017; Lubans & Morgan, 2008); two studies reported using both SCT and self-determination theory (Lubans et al., 2016; Owen et al., 2018). Other theories included diffusion of innovations theory (Sebire et al., 2018) and a youth empowerment approach (Aceves-Martins et al., 2017). Only one study did not report an underlying theory (Gobbi et al., 2018).

### Behavior Change Techniques

From all included studies in this review, four explicitly mentioned the BCTs employed in their intervention (Corder et al., 2016; Lubans et al., 2016; Owen et al., 2018; Sebire et al., 2018). All four studies reported PA improvements at the end of the study. Corder (2016) used the highest number of BCTs (*N* = 13) (Corder et al., 2016). Sebire (2018) and Owen (2018) both used 10 BCTs (Owen et al., 2018; Sebire et al., 2018), and Carlin et al. (2018) incorporated nine BCTs into their study. The top most frequently used BCTs were information about health consequences (67%), social support unspecified (56%), social support practical (56%), and demonstration of the behavior (56%). Details of frequency of use of BCTs are provided in Table 2 and a more detailed account of BCTs from all 18 included studies can be found in Supplemental Files 3 and 4. Social support ranged between unspecified, practical, and emotional social support and was reported in all nine studies with improved PA outcomes (Aceves-Martins et al., 2017; Carlin et al., 2018; Corder et al., 2016; Foley et al., 2017; Gobbi et al., 2018; Lubans et al., 2016; Lubans & Morgan, 2008; Owen et al., 2018; Sebire et al., 2018). The use of the BCT group comparison of behavior was reported through the use of BCTs demonstration of the behavior and social comparison in seven studies (Carlin et al., 2018; Corder et al., 2016; Foley et al., 2017; Gobbi et al., 2018; Lubans & Morgan, 2008; Owen et al., 2018; Sebire et al., 2018). Natural consequences was reported in six studies (Aceves-Martins et al., 2017; Carlin et al., 2018; Foley et al., 2017; Lubans et al., 2016; Owen et al., 2018; Sebire et al., 2018) using the BCT information about health consequences only. Goals and planning was reported in six studies using the BCTs goal setting (behavior), problem solving, goal setting (outcome), action planning, and review behavior goals (Carlin et al., 2018; Corder et al., 2016; Foley et al., 2017; Lubans et al., 2016; Owen et al., 2018; Sebire et al., 2018).

**Table 2.** Frequency of Behavior Change Techniques (BCTs) Used in the Studies (*n* = 9) With Improved Physical activity (PA) Outcome, Sorted by Most Commonly Appeared. If Numbers Were the Same, Then Ordered by BCTTv1 Number.

Behavior change technique label	*n* (%)
5.1 Information about health consequences	6 (67)
3.1 Social support (unspecified)	5 (56)
3.2 Social support (practical)	5 (56)
6.1 Demonstration of the behavior	5 (56)
1.1 Goal setting (behavior)	3 (33)
1.2 Problem-solving	3 (33)
10.1 Material incentive (behavior)	3 (33)
2.3 Self-monitoring of behavior	3 (33)
4.1 Instruction on how to perform the behavior	3 (33)
6.2 Social comparison	3 (33)
1.4 Action planning	2 (22)
10.2 Material reward (behavior)	2 (22)
10.4 Social reward	2 (22)
13.1 Identification of self as role model	2 (22)
2.1 Monitoring of behavior by others without feedback	2 (22)
3.3 Social support (emotional)	2 (22)
7.1 Prompts/cues	2 (22)
8.2 Behavior substitution	2 (22)
1.3 Goal-setting (outcome)	1 (11)
1.5 Review behavior goal(s)	1 (11)
10.5 Social incentive	1 (11)
10.8 Incentive (outcome)	1 (11)
12.2 Restructuring the social environment	1 (11)
14.9 Reduce reward frequency	1 (11)
2.2. Feedback on behavior	1 (11)
8.1 Behavioral practice/rehearsal	1 (11)

*Note*. BCTTv1 = behavior change technique taxonomy v1.

Information about health consequences was incorporated in six studies (Aceves-Martins et al., 2017; Carlin et al., 2018; Foley et al., 2017; Lubans et al., 2016; Owen et al., 2018; Sebire et al., 2018), social support (practical) (Aceves-Martins et al., 2017; Carlin et al., 2018; Gobbi et al., 2018; Lubans & Morgan, 2008; Owen et al., 2018), social support (unspecified) (Aceves-Martins et al., 2017; Corder et al., 2016; Foley et al., 2017; Lubans et al., 2016; Sebire et al., 2018), and demonstration of the behavior (Carlin et al., 2018; Corder et al., 2016; Foley et al., 2017; Gobbi et al., 2018; Lubans & Morgan, 2008) were incorporated in five studies, respectively.

### Training Provided for Peer Leaders

Four studies provided training for under two hours (Carlin et al., 2018; Corder et al., 2016; Gobbi et al., 2018; Lubans et al., 2016), 1 to 2 days (Foley et al., 2017; Sebire et al., 2018), two had consecutive sessions (Aceves-Martins et al., 2017; Owen et al., 2018), and one included a follow up session (Sebire et al., 2018). Table 3 shows a breakdown and a more detailed account of all included studies can be found in Supplemental File 5.

**Table 3.** Peer Leader Training Summary.

	Aceves-Martins et al. (2017)	Carlin et al. (2018)	Corder et al. (2016)	Foley et al. (2017)	Gobbi et al. (2017)	Lubans et al. (2008)	Lubans et al. (2016)	Owen et al. (2018)	Sebire et al. (2018)	*n*
Training provided	✓	✓	✓	✓	✓		✓	✓	✓	8
Length of training										
≤1 hour		✓	✓				✓			3
≤2 hours					✓					1
1–2 days				✓					✓	2
Consecutive sessions	✓							✓		2
Included follow up									✓	1
Training facilitator										
Researchers/university health specialist	✓	✓					✓		✓	4
University students				✓				✓		2
Teachers					✓					1
Intervention facilitator/coordinator			✓							1
Training content										
PA content	✓	✓					✓	✓	✓	5
Communication	✓				✓			✓	✓	4
Intervention content	✓						✓		✓	3
Practice delivery				✓	✓				✓	3
Content design	✓							✓		2
Ongoing support		✓	✓							2
Training manual		✓		✓						2

Peer-leadership training was facilitated by researchers/university health specialists in four studies (Aceves-Martins et al., 2017; Carlin et al., 2018; Lubans et al., 2016; Sebire et al., 2018), two used university students (Foley et al., 2017; Owen et al., 2018), one used teachers (Gobbi et al., 2018), and one used an intervention facilitator/coordinator (Corder et al., 2016). Positive PA outcomes were reported in studies with peer leaders who received a printed manual (Carlin et al., 2018; Foley et al., 2017). Training content provided in studies included communication skills (Aceves-Martins et al., 2017; Gobbi et al., 2018; Owen et al., 2018; Sebire et al., 2018), PA information (Aceves-Martins et al., 2017; Carlin et al., 2018; Lubans et al., 2016; Owen et al., 2018; Sebire et al., 2018), and intervention information (Aceves-Martins et al., 2017; Lubans et al., 2016; Sebire et al., 2018). Two studies reported ongoing support to peer leaders throughout the intervention (Carlin et al., 2018; Corder et al., 2016).

## Discussion

This systematic literature review examined the effectiveness of the peer-led approach to increase adolescents’ PA. It focused on identifying strategies deployed, BCTs, and peer-leadership training. Of the 18 included studies, nine reported improved PA outcomes, demonstrating that peer leadership can improve outcomes for PA levels when certain conditions are met. This evidence concurs with previous literature that peer leadership has the potential to be an effective way to influence PA behaviors in adolescents (Christensen et al., 2020; Hulteen et al., 2019). However, half of the studies did not report improvements on PA outcomes, illustrating that peer-led approaches are still underdeveloped, complex, and require high-quality evaluation techniques combined with implementation as intended.

All studies were in school-based settings, and this is an ideal setting for PA promotion due to the ease of reach of target populations (Kriemler et al., 2011). Implementation can be problematic given the importance of resource availability and quality in interventions (Naylor et al., 2015). Implementation time allocation issues reported in studies (Corder et al., 2016; Jenkinson et al., 2018) demonstrated the importance of infrastructure and policy support such as timetabling to allow peer leaders meet program demands (Daly-Smith et al., 2020) and provide for sustainable programs in the school setting (Lubans & Morgan, 2008).

Overall, 8/18 studies used measures to assess the extent to which peer-led components were implemented as originally intended (Carlin et al., 2018; Cui et al., 2012; Foley et al., 2017; Lubans et al., 2012; Lubans et al., 2016; Sebire et al., 2018; Smith et al., 2014; Tymms et al., 2016). Interactions between facilitators of peer-leadership training and peer leaders as well as between these peer leaders and peers is difficult to quantify, creating challenges to assess fidelity of implementation (Owen et al., 2018). With more flexible intervention designs that emphasize appropriate tools to assess fidelity in peer-led implementation, this would significantly advance knowledge from peer-led trials (Carroll et al., 2007; Wolfenden et al., 2021).

There were improved PA outcomes when peer leaders in mid-late adolescence led younger peers (Carlin et al., 2018; Corder et al., 2016; Foley et al., 2017; Owen et al., 2018) or peer leaders in early adolescence old led those of the same age (Aceves-Martins et al., 2017; Lubans et al., 2016; Lubans & Morgan, 2008; Sebire et al., 2018). Overall, those in early adolescence had less success in peer leadership in the secondary school setting. The identification of the appropriate age for peer leadership is an important characteristic that should be considered when appointing peer leaders (Christensen et al., 2020). The majority of studies targeted students aged 11 to 15 years and with the exception of one study, adolescents aged 16 years and older have yet to be the targeted population in the school setting (Gobbi et al., 2018). There are also a limited number of PA promoting interventions and limited evidence on strategies to best promote PA in older adolescents in schools (Hynynen et al., 2016). Future research is required to target older adolescents (Hartwig et al., 2021) to ascertain the ways in which we can establish behavior change in this age cohort examining whether the peer-led approach is an appropriate mechanism. Future research is required to ascertain the ways in which we can establish behavior change in this age cohort (Hartwig et al., 2021) and to determine whether the peer-led approach represents an appropriate mechanism.

In studies that targeted solely PA behavior, 6/9 studies had improved PA outcomes. This was in contrast with 7/9 studies not reporting improved PA outcomes that only targeted PA as a component of the intervention. Aiming to target more than one behavior, for example, healthy eating and PA can be multidimensional thus assisting in complexities of behavior change, particularly through adolescent peer networks (Bell et al., 2017). However, targeting PA through peer leadership in the school setting also improved social connectedness, well-being, and peer social support (Corder et al., 2016; Jenkinson et al., 2018; Owen et al., 2018; Tymms et al., 2016). Peer leadership encourages mutually beneficial interaction and support for both peer leaders and peers (Huang et al., 1995). Two studies measured PA among peer leaders and reported increases regardless of peer leader gender (Owen et al., 2018) and in boy peer leaders only (Foley et al., 2017). This is in line with previous reviews which suggested additionally measuring outcomes for peer leaders while also measuring changes in leadership and education-enhancing behaviors (Hulteen et al., 2019; Jenkinson et al., 2013). Future research into the peer-led approach in PA promotion should establish the mutual benefits for both peers and peer leaders as well as establish physical, social, and mental health benefits beyond PA outcomes.

Psychological theories underpinned 8/9 studies with improved PA, with SCT used most frequently (Bandura, 1986). In those studies, role modelling and increased self-efficacy were the intended mechanisms for behavior change. Two of the three studies underpinned by youth empowerment theory demonstrated improved outcomes for PA (Aceves-Martins et al., 2017; Foley et al., 2017). Empowering youth is a relatively new phenomenon (Morton & Montgomery, 2013) and involves providing both leaders and participants with meaningful input into the development and running of interventions through gaining student voice (Mitra, 2018). Diffusion of innovations theory also showed promise for influencing behavior change through peer nomination of leaders to disseminate messages to their peers informally through encouragement, sharing knowledge, and co-participation (Rogers, 1983). The study by Tymms et al. (2016) acknowledged the need to consider broader and complex ecosystemic influences to increase PA behaviors in children (Bronfenbrenner, 2005; Tymms et al., 2016). For future development of peer-led interventions, the use of role modelling, peer nomination, and youth empowerment underpinned by theory is suggested.

Based on the evidence from this review, peer leadership in secondary-level schools was effective when the intervention was targeted at adolescent girls (Carlin et al., 2018; Owen et al., 2018; Sebire et al., 2018). The study by Lubans et al. (2016) also specifically targeted boys with positive intervention effects for PA. Of the four mixed-gender studies with improved PA outcomes, one reported only male peer leaders had significant increases in MVPA compared with a decline in girl’s MVPA (Foley et al., 2017). In the study by Aceves-Martins et al. (2017), boys also showed more interest in PA promoting activities compared with girls who took more interest in the nutrition activities (Aceves-Martins et al., 2017). This highlights that one size does not fit all when considering appropriate activities for mixed-gender schools and the need to implement-specific strategies to engage students (Gibbons & Naylor, 2007). Empowering young people in the design and implementation of interventions and facilitating student voice can be an efficient mechanism for identifying specific activity needs of students (Aceves-Martins et al., 2017; Mitra, 2018).

Hulteen et al. (2019) suggested that future research should aim to identify appropriate BCTs to be employed in order to optimize PA behavior change in peer-led interventions. The four studies that had the highest frequency of BCTs (9–13 BCTs) reported improved PA outcomes (Carlin et al., 2018; Corder et al., 2016; Owen et al., 2018; Sebire et al., 2018). This is in line with previous literature on adolescent PA and sedentary behavior whereby the effective studies had more BCTs compared with ineffective studies (Hendrie et al., 2012; Hynynen et al., 2016; Schoeppe et al., 2017). Commonly reported BCTs across these studies were material incentive and goal setting (behavior) (Carlin et al., 2018; Corder et al., 2016; Owen et al., 2018), information about health consequences (Carlin et al., 2018; Owen et al., 2018; Sebire et al., 2018), and social comparison (Corder et al., 2016; Owen et al., 2018; Sebire et al., 2018). Three out of these four studies targeted girls only (Carlin et al., 2018; Owen et al., 2018; Sebire et al., 2018) and three out of the four studies also deployed older peer leaders in mid-late adolescence (Carlin et al., 2018; Corder et al., 2016; Owen et al., 2018). Our review found that social support techniques were deployed by all studies that reported an improved PA outcome as also found in previous reviews (Hendrie et al., 2012; Hynynen et al., 2016), reinforcing the support of peers as essential in the adoption and maintenance of PA behaviors (Lawler et al., 2020). The evidence for including social support in promoting adolescent’s PA is apparent and fits with theoretical models such as SCT (Bandura, 1986) and expectancy-value theory (Eccles & Wigfield, 2002). Although social support alone is insufficient, as effective peer-led interventions combined demonstration of behavior (Brannon & Cushing, 2015; Hsu et al., 2018; Schoeppe et al., 2017) or goal setting (Brannon & Cushing, 2015) and the use of information about health consequences as effective BCTs (Hendrie et al., 2012; Hynynen et al., 2016).

All but one study in this review reported training for peer leaders, demonstrating its importance for peer-led programs (Story et al., 2002). In all but one study, the intervention was delivered by personnel from external intervention facilitators. Improved PA outcomes were reported where peer leaders were provided with a training manual (Carlin et al., 2018; Foley et al., 2017). With changes in technology, future interventions may need to provide peer leaders with digital versions of training content through the use of apps, social media, remote classrooms, and so on (Brannon & Cushing, 2015; Hsu et al., 2018). Additionally, the use of apps for participants to monitor behavior (Brannon & Cushing, 2015) to replace physical reward cards (stamped each time a walk was completed) as reported in Carlin et al.’s (2018) study may have potential given schools have found themselves engaging with students remotely due to COVID-19 restrictions (Ng et al., 2020).

Our review found advantages in the use of various methods of appointing peer leaders either through volunteering, peer nomination, or selection by teachers (Carlin et al., 2018; Lubans et al., 2012; Lubans et al., 2016; Lubans & Morgan, 2008; Smith et al., 2014; Tymms et al., 2016). Where students volunteered, it facilitated inclusiveness instead of electing already respected peers in the class, to build capacity (Foley et al., 2017). Finding the appropriate leaders or influential adolescents can act as change agents throughout social networks in the school and classroom (Valente, 2012; van Woudenberg et al., 2018). Peers selected through peer nomination play a vital role to share knowledge, provide encouragement, and support as well as engage in co-participation and shifting norms (Sebire et al., 2018) and students can implement BCTs such as modelling or social support more effectively than if the target group do not identify with the person modelling the behavior (Schoeppe et al., 2017). This can lead to a rotation of roles and responsibilities among the students (Story et al., 2002). Although peer nomination has its logistical challenges, a mechanism for such an approach is the concept of shared leadership (Mertens et al., 2020), and warrants further study of such processes in peer-led PA interventions. More research is needed on the use of either students volunteering, peer nomination or selection by teacher to ascertain the most effective methods of peer leader selection.

## Strengths and Limitations

The use of the BCTTv1 to map BCTs used in peer-led programs is a strength of this study (Michie et al., 2013). These studies were also assessed for methodological quality using a modified tool (van Sluijs et al., 2007). This review was limited whereby only studies in English language were eligible for inclusion, potentially excluding relevant evidence. There were different measures of PA adopted across the included studies, such as questionnaires, accelerometers, and pedometers. Many studies included peer-led components as one of a multi-component intervention. Few studies explored the process evaluation and effectiveness solely from peer-led strategies and robust evaluations of targeted components in multi-component designs are needed.

## Implications for Research, Theory, and Practice

This systematic review has identified BCTs that may be effective in adolescent peer-led PA promoting interventions. Social support (practical and unspecified), information about health consequences, and demonstration of the behavior by peer leaders are promising BCTs. Use of modelling, empowerment, and popular opinion leaders, underpinned by theory has also shown potential. Younger adolescents can be effectively led by both older and same age peers; however, there is a dearth of PA promoting programs targeting older adolescents. Careful consideration is required when identifying the frequency, variance, and relevance of BCTs. The variability in type, length, and method of training for peer leaders identified in this review highlights the need for a more comprehensive outline of training provided to peer leaders and should be investigated further to guide future work in this area. Moreover, the method in the selection of peer leaders should also gain student opinion to guide this process. The majority of studies did not report the active ingredients used and streamlined reporting of interventions targeting similar groups would enhance understanding among researchers and intervention developers about what works and how (Duff et al., 2017; Hynynen et al., 2016). There are a variety of peer-to-peer approaches where peers can support peers. This variety of approaches and need for streamlined reporting of interventions lends itself to the potential development of a typology, conceptual framework, and practice guidelines for peer-led strategies for the adolescent population (Matz-Costa et al., 2019). This conceptualization of the delivery and interactions between peer leaders and participating peers as well as design characteristics of the peer–peer dynamic, the setting, modality, level of formality, and the peer assignment strategy would, along with relevant BCTs and training strategies guide future peer-led interventions in all relevant settings for adolescents (Matz-Costa et al., 2019). Regardless of the approach taken, peer leadership, undertaken in the school setting within a full social ecological model (Bronfenbrenner, 1986) can be a resourceful way of promoting PA in adolescents.

## Supplementary Material

Supplementary material

Supplementary material

Supplementary material

Supplementary material

Supplementary material
