# Supplementary material for: A Systematic Literature Review of Peer-led Strategies for Promoting Physical Activity Levels of Adolescents
Source: Health Educ Behav. 2021 Oct 11;49(1):41–53. doi: 10.1177/10901981211044988 (PMC8892039; doi:10.1177/10901981211044988)
Supplement: Supplementary material [file sj-docx-1-heb-10.1177_10901981211044988.docx]

*Quality of studies assessment*

| **Study ref** | **A** | **B** | **C** | **D** | **E** | **F** | **G** | **H** | **I** | **J** | **High, Low, Medium** |
| --- | --- | --- | --- | --- | --- | --- | --- | --- | --- | --- | --- |
| Aceves-Martins et al. (2017) | + | + | + | + | + | ? | ? | + | + | + | 8 (High) |
| Bell et al. (2014) | + | N/A | + | + | + | ? | N/A | + | + | ? | 6 (High) |
| Carlin et al. (2018) | ? | + | + | + | + | + | ? | + | + | + | 8 (High) |
| Corder et al. (2016) | ? | + | + | + | - | + | + | - | + | + | 7 (High) |
| Cui et al. (2012) | - | + | + | + | + | + | + | + | - | + | 8 (High) |
| Foley et al. (2017) | N/A | N/A | + | + | ? | N/A | N/A | ? | N/A | ? | 2 (Medium) |
| Gobbi et al. (2017) | N/A | N/A | + | + | + | N/A | N/A | N/A | - | - | 3 (Medium) |
| Haapala et al. (2017) | + | N/A | + | + | - | - | N/A | + | ? | ? | 4 (Medium) |
| Jenkinson et al. (2018) | + | N/A | + | + | + | + | N/A | - | ? | ? | 5 (High) |
| Lubans et al. (2008) | + | N/A | + | - | N/A | + | N/A | + | - | - | 5 (Medium) |
| Lubans et al. (2012) | + | + | + | + | + | + | - | + | + | ? | 8 (High) |
| Lubans et al. (2011) | + | + | + | - | - | + | - | + | + | ? | 6 (High) |
| Lubans et al. (2016) | + | + | + | + | + | + | - | + | + | ? | 8 (High) |
| Owen et al. (2018) | + | N/A | + | + | - | + | N/A | - | + | + | 6 (High) |
| Sebire et al. (2018) | + | + | + | + | + | + | + | + | + | + | 10 (High) |
| Smith et al. (2014) | ? | + | ? | + | ? | + | - | ? | + | + | 5 (Medium) |
| Tymms et al. (2016) | - | + | + | + | - | + | - | - | ? | + | 5 (Medium) |
| Utter et al. (2011) | + | + | - | ? | - | + | - | + | ? | + | 5 (Medium) |

NOTES: Description of columns: A: Groups comparable; B: Randomisation; C: Unit of analysis; D: PA measure; E: Drop out; F: Timing; G: Blinding; H: Follow-up; I: Intention-to-treat; J: Confounders. Possible scores obtained: ‘+’: positive; ‘?’: not or insufficiently described; ‘-‘: negative.

RCTs defined quality as high when given a score of 6 or more, medium when scored 4 or more and low when scored less than 4.

Non RCT defined quality as high when given a score of 5 or more, medium when scored 3 or more and low when scored less than 3

All other studies defined quality as high when given a score of 4 or more, medium when scored 2 or more and low when scored less than 2.

| **Item** | **Description** |
| --- | --- |
| A | Groups comparable at baseline on key characteristics (positive if stratified baseline characteristics were presented for age, sex, and at least one relevant outcome measure; for cluster randomised controlled trials and controlled trials, positive if this was statistically tested |
| B | Randomisation procedure clearly described and adequately carried out |
| C | Unit of analysis was individual (negative if unit of analysis was school level or school level randomisation not accounted for in  individual level analyses) |
| D | Validated measures of physical activity used (positive if validation of measures of physical activity was reported or referred to) |
| E | Dropout described and not more than 20% for studies with follow-up of six months or shorter and 30% for studies with follow-up of more than six months |
| F | Timing of measurements comparable between intervention and control groups |
| G | Blinding outcome assessment (positive if those responsible for assessing physical activity at outcome were blinded to group  allocation of individual participants) |
| H | Participants followed up for a minimum of six months |
| I | Intention to treat analysis used |
| J | Potential confounders accounted for in analyses |

Tool used by Sluijs, 2007
