# Supplementary material for: A Systematic Literature Review of Peer-led Strategies for Promoting Physical Activity Levels of Adolescents
Source: Health Educ Behav. 2021 Oct 11;49(1):41–53. doi: 10.1177/10901981211044988 (PMC8892039; doi:10.1177/10901981211044988)
Supplement: Supplementary material [file sj-docx-2-heb-10.1177_10901981211044988.docx]

*Summary extraction table*

| **Author and country** | **Intervention Name and theoretical framework** | **Study design and duration** | **Full peer-led intervention** | **Peer-led description** | **Age dynamic of Peers/peer leaders and gender of populations** | **Method of choosing mentors** | **PA Measurement and outcome** |
| --- | --- | --- | --- | --- | --- | --- | --- |
| Aceves-Maetins et al. (2017)  Spain | "We Are Cool”—Som la Pera Study (EYTO project-The European Youth Tackling Obesity)  *Youth Empowerment* | Parallel cluster randomised controlled trial  12 months across 2 academic years | **✓** | 5 ACCs (Adolescent challenge creators) designed and implemented 10 activities as challenges for their peers. Presented the intervention in 11 classrooms explaining the study, providing social media information, and invited their peers to provide suggestions for activities. Disseminated the activities using social media platforms as well as posters and  flyers. | Peers: 13-16 years (mean:14.66–0.77) Peer leaders: Same age  Mixed | Selected by teachers (Based on their knowledge of the students’ leadership  characteristics and English language skills-to facilitate  communication with EYTO partners) | *Health Behaviour in Schoolchildren (HBSC) survey*  Intervention adolescents showed an increase of 18.5% in ≥6 hours/week of PA (p < 0.01) compared with controls. |
| Bell et al. (2014)  England | Activity and healthy Eating in Adolescence (AHEAD)  *Diffusion of innovations theory* | Exploratory trial  1 academic year | **✓** | 17% (79) students became "peer supporters" Informal diffusion of health messages. Included cooking demonstration led by peers. Physical activities-skipping, circus skills and Frisbee | Peers: 12-13 years Peer leaders: Same age  Mixed | Peer nomination | *Accelerometer*  No difference between MVPA in intervention and control groups. |
| Carlin et al.  (2018)  Northern Ireland | Walking in Schools intervention (the WISH study)  *Social Cognitive theory* | Cluster randomised controlled trial  12 weeks | **✓** | 10-15 minutes peer-led brisk walks throughout the school week. | Peers: 12-13 years  Peer leaders: 15-17 years  Girls | Recruitment not reported | *Accelerometer (ActiGraph GT3)*  Intervention group increased light intensity PA by 8.27 minutes per day compared with a decrease of 2.14 mins/day in control group (p = 0.003. |
| Corder et al.  (2016)  England | GoActive  *Social Cognitive Theory* | Feasibility study and pilot cluster-randomised controlled trial  8 weeks | **✓** | Older adolescent mentors (2 per class group) encouraged participation in activities and helped by year 9 in-class peer leaders who change weekly.  Students gain points for trying activities which are entered into an intramural competition. GoActive aims to increase PA through increased peer support, self-efficacy, self-esteem, group cohesion and friendship quality, and is implemented in tutor groups using a tiered leadership system | Peers: 13-14 years  Peer leaders: 15-18 years  Mixed | Nominated by the school | *Accelerometer (ActiGraph GT1M or GT3X)*  5.1 min/day difference in MVPA in favour of intervention group (p=0.014) |
| Cui et al. (2012)  China | *Social cognitive theory & Empowerment educational approach* | Pilot study  4 weeks | **✓** | 4 components: food choice, physical activity and sedentary behaviour, carbonated drinks and goal setting, Peer leaders delivered four 40-min peer education lessons to their classmates over four consecutive weeks in their classrooms, following the peer leader’s manual. The four peer education activities were integrated into the existing health education courses and class meetings. Lessons included presentation, video watching, group discussion, games experiments, lifestyle practice, skit playing and quiz show. | Peers: Grade 7  Peer leaders: Same age  Mixed | Selected by class teacher from volunteer students based on organisation, oral expression ability, influence among students and sense of responsibility. | A validated 7-day youth PA questionnaire (modified to collect MVPA and SB in previous week)  No significant difference in time  in MVPA between  intervention and control group |
| Foley et al.  (2017)  Australia | Students As LifeStyle Activists (SALSA)  *Social Cognitive theory*  *& Empowerment education approach* | Pre-post study design  4x 70 min lessons (overall length not reported - measurements taken immediately before training workshop and two-weeks after after final lesson) | **✓** | The SALSA lessons delivered by between four and six peer leaders per group, cover healthy eating, physical activity and living a healthy lifestyle using alternative methods of learning including a video, games, roleplaying and a quiz show. The four, 70-min SALSA lessons are designed to integrate with the Personal Health, Development and Physical Education (PDHPE) curriculum. | Peers: 13-14 years  Peer leaders: 15-16 years  Mixed | Volunteered  or were selected by teachers | *Self-report questionnaire*  14.2% change in boys meeting MVPA levels pre -post (6.9–21.5)  No significant change in girls meeting MVPA levels pre-post |
| Gobbi et al.  (2017)  Italy | Peer tutored Physical Education (PTPE)  *Not reported* | Mixed model design  1 academic year | **✓** | Students with mild to moderate intellectual disabilities buddied with typically developing peer within an extra 1 hour PE class once a week delivered immediately after curricular school hours. | Peers: 15-19 years  Peer leaders: Same age  Mixed | Volunteered | *Accelerometer (ActiGraph GT3X)*  Participants reported higher light intensity PA (*p* < .001) |
| Haapala et al. (2017)  Finland | Finnish Schools on the move  *Social ecological model* | Quasi-experimental design  2 academic years | x | Students as “recess activators”/peer instructors at break times helping the hired project worker. Also involved in planning activities. | Differed depending on the school.  Same age and/or mentors older age (ages not specified)  Mixed | Recruitment by researchers | *Accelerometer (ActiGraph GT1M*  *and GT3X)*  The proportion  of males who had at least 5 active days per  week (with>60 min of MVPA per day) decreased  from 51% to 44%. The trend was similar in females  (decline from 45% to 40%) |
| Jenkinson et al. (2018)  Australia | GLAMA (Girls! Lead!  Achieve! Mentor! Activate!) and BLAST (Boys!  Lead! Activate! Succeed Together!)  *Social Cognitive Theory* | Non-randomised controlled trial  8 weeks | **✓** | Induction into school life incorporating activities led by older mentors | Peers: 10-12 years  Peer leaders: 15 years (intervention school requested to be gender matched)  Boys | Potential leaders completed  written applications, with suitable leaders then selected by two teachers | *Self-Report questionnaire*  No significant school effects reported for number of days of completing 60  minutes of physical activity were observed |
| Lubans et al.  (2008)  Australia | LEAF (Learning to Enjoy  Activity with Friends’)  *Social Cognitive Theory* | Quasi experimental design  8 weeks | x | Being active with friends” Modelling (students trained with partners) and provided social support from friends. | Peers: 14-15 years  Peer leaders: Same age  Mixed | Recruitment not reported | *Pedometer*  Low-active adolescents (intervention group) at post-test in the  Increased physical activity by 2341 steps/day, a  significant increase from baseline measures (P<0.05) and significantly different to that in  the comparison group (P<0.05). |
| Lubans et al.  (2012)  Australia | NEAT Girls-Nutrition and Enjoyable Activity for Teen Girls  *Social Cognitive Theory* | Group Randomised controlled trial  12 months | x | Weekly lunchtime PA sessions complementing school sport sessions, organized and delivered by girls | Peers: 12-14 years  Peer leaders: Same age  Girls | Recruitment not reported | *Accelerometer (ActiGraph GT1M*  *and GT3X)*  No group by time effects for physical activity |
| Lubans et al.  (2011)  Australia | PALs program-Physical Activity Leaders  *Self-determination theory &*  *Social cognitive Theory* | Randomised Controlled Trial  6 months | **✓** | Interactive seminars, lunch time activities, leadership sessions. Gained PALs accreditation by delivering the above to grade 7 students under supervision of teachers. | Peers: Grade 7 (11-12-years) Peer leaders: Grade 9 (13-14 years)  Boys | Chosen by PE teachers (identified low-active boys considered to be disengaged in PE and/or not currently participating in individual/team sports) | *Pedometers*  No Significant results |
| Lubans et al.  (2016)  Australia | ATLAS-Active Teen leaders avoiding screen time  *Self-determination theory &*  *Social cognitive Theory* | Cluster Randomised controlled trial  20 weeks | x | 20 minutes lunch-time physical activity leadership sessions run by students | Peers: 12-14 years  Peer leaders: Same age  Boys | Recruitment not reported | Accelerometer (ActiGraph GT3X)  Intervention group classified as low-active at baseline increased their step counts (baseline: 7716 steps/day, post-test: 10,301 steps/day, and accumulated significantly more steps than their peers |
| Owen et al.  (2018)  England | G-PACT-Girls Peer  Activity  *Social Cognitive Theory &*  *Self Determination theory* | 3-arm, parallel group, non-randomised feasibility trial  7 weeks | **✓** | Encouraged peers to be more physically active and social support role & designed posters | Peers: 13-15 years  Peer leaders: 15-16 years  Girls | Selected by PE teachers based on leadership abilities, communication skills, potential role model, confidence and social influence. | *Accelerometer (ActiGraph GT9X)*  Time x school effect (p = 0.012) for leaders’ whole day MVPA levels  time x school effect for Peers’ school day MVPA (p < 0.001)    “Class” peers- Significantly increased whole day MVPA by 3.2 min  “Choice” peers-A significant main effect for time in after school club but not for MVPA.  “No Club” peers-significantly decreased MVPA by 3min |
| Sebire et al.  (2018)  England | PLAN-A  *Diffusion of innovations theory* | A two-arm cluster randomised controlled feasibility study  1 academic year | **✓** | Informally diffused messages and norms about leading physically active lifestyles to their friends. | Peers: 12-13 years  Peer leaders: Same age  Girls | Questionnaire completed to identify the influential girls in their school. | *Accelerometer (ActiGraph GT3X)*  6.09 mins of MVPA difference between groups in favour of intervention arm |
| Smith et al.  (2014)  Australia | ATLAS- Active Teen leaders avoiding screen time  *Self Determination Theory &*  *Social Cognitive Theory* | Cluster Randomised controlled trial  20 weeks | x | Lunchtime sessions instructing on use of resistance bands. Smart phone app for peer assessment | Peers: 12-13 years  Peer leaders: 13-14 years  Boys | Recruitment not reported | *Accelerometer (ActiGraph GT3X)*  No significant intervention effects for PA (mean counts per minute or MVPA) |
| Tymms et al.  (2016)  England | The MOVE project  *Not reported* | Cluster Randomised controlled trial  10 months | **✓** | Children paired up 1:1 with peer mentor for 6 weekly mentoring meetings of 20-30 mins . Provided learning support to partner and facilitated critical reflection on his/her PA habits. Goal setting | Peers: 11 years  Peer leaders: 13-14 years  Mixed | Recruitment not Reported | *Accelerometer (ActiGraph GT1M, GT3X and GT3X+)*  No significant effects (main or interaction)  were observed for PA |
| Utter et al.  (2011)  New Zealand | Living 4 life project  *Youth Development Aotearoa Strategy* | Quasi experimental  3 years | **✓** | Breakfast clubs, lunch-time activities, after-school dance, health weeks designed and run by students (Student health councils) | Peers: 9-13 years  Peer leaders: Same age  Mixed | Voluntary-(invited to take part on Student health councils weekly) | *Self Report questionnaire*  Lunchtime and after school activity the last 5days: No significant changes on lunchtime or after school activity in any schools. |
