# Supplementary material for: A Systematic Literature Review of Peer-led Strategies for Promoting Physical Activity Levels of Adolescents
Source: Health Educ Behav. 2021 Oct 11;49(1):41–53. doi: 10.1177/10901981211044988 (PMC8892039; doi:10.1177/10901981211044988)
Supplement: Supplementary material [file sj-docx-3-heb-10.1177_10901981211044988.docx]

*BCTs used in the included studies*

|  | Goals & planning  1.1-1.9 | | | | | Feedback & monitoring  2.1-2.7 | | | Social support  3.1-3.3 | | | Shaping knowledge  4.1-4.4 | Natural consequences  5.1-5.6 | Comparison of behaviour  6.1-6.3 | | Associations  7.1-7.8 | Repetition & substitution  8.1-8.7 | | Reward & Threat  10.1-10.11 | | | | | | Antecedents  12.1-12.6 | | Identity  13.1-13.5 | Scheduled consequences  14.1-14.10 |  |
| --- | --- | --- | --- | --- | --- | --- | --- | --- | --- | --- | --- | --- | --- | --- | --- | --- | --- | --- | --- | --- | --- | --- | --- | --- | --- | --- | --- | --- | --- |
|  | **1.1** | **1.2** | **1.3** | **1.4** | **1.5** | **2.1** | **2.2** | **2.3** | **3.1** | **3.2** | **3.3** | **4.1** | **5.1** | **6.1** | **6.2** | **7.1** | **8.1** | **8.2** | **10.1** | **10.2** | **10.4** | **10.5** | **10.6** | **10.8** | **12.2** | **12.5** | **13.1** | **14.9** |  |
| *Aceves-Maetins et al 2017 #1 |  |  |  |  |  |  |  |  | ✓ |  |  |  | ✓ |  |  |  | ✓ |  |  |  |  |  |  |  |  |  |  |  | 3 |
| Bell et al 2014 #2 | ✓ | ✓ |  |  | ✓ |  |  |  | ✓ |  |  |  | ✓ |  |  |  |  | ✓ |  |  |  |  |  |  |  |  |  |  | 6 |
| *Carlin et al. 2018 #3 | ✓ |  |  |  |  | ✓ |  | ✓ |  | ✓ |  | ✓ | ✓ | ✓ |  | ✓ |  |  | ✓ |  |  |  |  |  |  |  |  |  | 9 |
| *Corder et al 2016 #4 | ✓ |  |  |  |  |  |  |  | ✓ |  |  | ✓ |  | ✓ | ✓ |  |  |  | ✓ | ✓ | ✓ | ✓ |  | ✓ | ✓ |  | ✓ | ✓ | 13 |
| Cui et al 2012 #5 | ✓ |  |  |  |  |  |  |  | ✓ |  |  |  | ✓ |  |  |  |  |  |  |  |  |  |  |  |  |  | ✓ |  | 4 |
| *Foley, 2017 #6 |  | ✓ |  |  |  |  |  |  | ✓ | ✓ |  |  | ✓ | ✓ |  |  |  | ✓ |  |  |  |  |  |  |  |  |  |  | 6 |
| *Gobbi et al. 2017 #7 |  |  |  |  |  |  |  |  |  | ✓ |  |  |  | ✓ |  |  |  |  |  |  |  |  |  |  |  |  |  |  | 2 |
| Haapala et al 2017 #8 |  |  |  |  |  |  |  |  |  | ✓ |  |  |  | ✓ |  |  |  |  |  |  |  |  |  |  |  |  |  |  | 2 |
| Jenkinson et al 2018 #9 |  |  |  |  |  |  |  |  | ✓ | ✓ |  |  | ✓ | ✓ |  |  |  |  |  |  | ✓ |  | ✓ |  |  |  |  |  | 6 |
| *Lubans et al. 2008 #10 |  |  |  |  |  |  |  |  |  | ✓ | ✓ |  |  | ✓ |  |  |  |  |  |  |  |  |  |  |  |  |  |  | 3 |
| Lubans et al 2012 #11 |  |  |  |  |  |  | ✓ | ✓ |  | ✓ |  |  | ✓ | ✓ |  |  |  |  |  |  |  |  |  |  |  |  | ✓ |  | 6 |
| Lubans et al. 2011 #12 |  | ✓ |  |  |  |  | ✓ | ✓ |  | ✓ |  |  | ✓ | ✓ |  |  |  |  |  |  |  |  |  |  |  |  | ✓ |  | 7 |
| *Lubans et al 2016 #13 |  | ✓ |  |  |  |  |  | ✓ | ✓ |  |  | ✓ | ✓ |  |  |  |  |  |  |  |  |  |  |  |  |  |  |  | 5 |
| *Owen et al 2018 #14 | ✓ | ✓ |  | ✓ | ✓ |  | ✓ | ✓ |  | ✓ |  |  | ✓ |  | ✓ | ✓ |  |  |  |  |  |  |  |  |  |  |  |  | 10 |
| *Sebire et al 2018 #15 |  | ✓ | ✓ | ✓ |  |  |  |  | ✓ |  | ✓ |  | ✓ |  | ✓ |  |  |  | ✓ | ✓ |  |  |  |  |  |  | ✓ |  | 10 |
| Smith et al. 2014 #16 | ✓ |  |  |  |  | ✓ | ✓ | ✓ |  |  |  | ✓ |  | ✓ | ✓ |  |  |  |  |  |  |  |  |  |  |  |  |  | 7 |
| Tymms et al 2016 #17 | ✓ | ✓ |  |  | ✓ |  |  | ✓ |  | ✓ | ✓ |  |  |  |  |  |  |  |  |  |  |  |  |  |  |  |  |  | 6 |
| Utter et al 2011 #18 |  |  |  |  |  |  |  |  |  | ✓ |  |  |  |  |  |  |  |  |  |  |  |  |  |  |  | ✓ | ✓ |  | 3 |

*Studies reporting on improved PA outcomes
