# Supplementary material for: A Systematic Literature Review of Peer-led Strategies for Promoting Physical Activity Levels of Adolescents
Source: Health Educ Behav. 2021 Oct 11;49(1):41–53. doi: 10.1177/10901981211044988 (PMC8892039; doi:10.1177/10901981211044988)
Supplement: Supplementary material [file sj-docx-4-heb-10.1177_10901981211044988.docx]

*Frequency of BCTs, sorted by most common used in 18 included studies and examples provided*

| **BCT label** | **BCT group** | **E.g. of how the BCT was used** | **Frequency of use** | **Studies where found (#)** |
| --- | --- | --- | --- | --- |
| **3.2 Social support (practical)** | Social support | “…Team of peers along with leader took part in challenges together…” #9 | 11 (61%) | 3, 6-12, 14, 17-18 |
| **5.1 Information about health consequences** | Natural consequences | “…Information booklet provided to students and their parents…” #5 | 11 (61%) | 1-3, 5-6, 9, 11-15 |
| **6.1 Demonstration of the behaviour** | Comparison of behaviour | “…Students participated in self-directed physical activity sessions involving elastic tubing resistance training…” #12 | 10 (56%) | 3-4, 6-12, 16 |
| **3.1 Social support (unspecified)** | Social support | “…Peer leaders trained to actively interact with peers and to facilitate interaction between peers…” #5 | 8 (44%) | 1-2, 4-6, 9, 13, 15 |
| **1.1 Goal-setting (behaviour)** | Goals and planning | “…Students are encouraged to set goals to increase their daily step counts…” #16 | 7 (39%) | 2-5, 14, 16-17 |
| **1.2 Problem-solving** | Goals and planning | “…Peer mentors identified barriers to PA with mentees and facilitated critical reflection by the mentee on his/her PA habits and how these could be improved…” #17 | 7 (39%) | 2, 6, 12-15, 17 |
| **2.3 Self-monitoring of behaviour** | Feedback and monitoring | “…Mentee was encouraged to self-monitor their progress using an activity diary…” #17 | 7 (39%) | 3, 11-14, 16-17 |
| **13.1 Identification of self as role model** | Identity | “…self-reflection on personal skills and interests which may make them a good peer-supporter…” #15 | 6 (33%) | 4-5, 11-12, 15, 18 |
| **2.2. Feedback on behaviour** | Feedback and monitoring | “…Smart phone app and website for peer assessment of resistance training exercises…” #16 | 4 (22%) | 11-12, 14, 16 |
| **4.1 Instruction on how to perform the behaviour** | Shaping knowledge | “…Peer leaders emphasised the importance of the walks being performed at a brisk pace for MPVA…” #3 | 4 (22%) | 3-4, 13, 16 |
| **6.2 Social comparison** | Comparison of behaviour | “...interactive tasks to find out what counts as PA PA recommendations and levels of PA in adolescent girls**…” #**15 | 4 (22%) | 4, 14-16 |
| **1.5 Review behaviour goal(s)** | Goals and planning | “…During subsequent weeks, the pair reviewed goal progress…” #17 | 3 (17%) | 2, 14, 17 |
| **10.1 Material incentive (behaviour)** | Reward and threat | “…A reward card stamped by a walk leader on completion of a walk for entrance into a draw to win small tokens…” #3 | 3 (17%) | 3-4, 15 |
| **3.3 Social support (emotional)** | Social support | “…interactive games to highlight key skills related to listening to peers about being active…” #15 | 3 (17%) | 10, 15, 17 |
| **1.4 Action planning** | Goals and planning | “…Peer leaders were taught how to set effective goals and develop goal setting skills / set targets to achieve…” #14 | 2 (11%) | 14-15 |
| **10.2 Material reward (behaviour)** | Reward and threat | “…Students will be informed of the GoActive reward system…” #4 | 2 (11%) | 4, 15 |
| **2.1 Monitoring of behaviour by other without feedback** | Feedback and monitoring | “…Pedometer step counts can also be entered into the smartphone app for review…” #16 | 2 (11%) | 3, 16 |
| **7.1 Prompts/cues** | Associations | “…Weekly verbal reminders to attend the walking sessions from school staff and walk leaders…” #3 | 2 (11%) | 3, 14 |
| **8.2 Behaviour substitution** | Repetition and substitution | “…Sedentary behaviour (before) changed to active behaviour (after), e.g. taking the lift changed to walking the stairs…” #2 | 2 (11%) | 2, 6 |
| **1.3 Goal-setting (outcome)** | Goals and planning | “…Goal setting: learning how to set ‘SMART goals’ and planning two peer-supporter/activity goals…” #15 | 1 (6%) | 15 |
| **10.4 Social reward** | Reward and threat | “…Points were accumulated throughout the program and winning teams were announced at the school assembly after completion of the program…” | 1 (6%) | 4, 9 |
| **10.5 Social incentive** | Reward and threat | “…Students are informed that verbal praise will be provided…” #4 | 1 (6%) | 4 |
| **10.6 Non-specific incentive** | Reward and threat | “…Team points were awarded for completion of challenges within time limits, scoring a certain number of points, completing tasks cooperatively, and staff were also involved in providing bonus points...” #9 | 1 (6%) | 9 |
| **10.8 Incentive (outcome)** | Reward and threat | “…Rewards are given out in front of peers; trophy awards (e.g., Development Award) are handed out at full year assembly at intervention end…” #4 | 1 (6%) | 4 |
| **12.2 Restructuring the social environment** | Antecedents | “…A regular short (∼20 min) intervention session is incorporated into the school timetable…” #4 | 1 (6%) | 4 |
| **12.5 Adding objects to the environment** | Antecedents | “…SHCs (Peer leaders) staffed the equipment library for students to sign out equipment and arranged for external activity providers…” #18 | 1 (6%) | 18 |
| **14.9 Reduce reward frequency** | Scheduled consequences | “…Students receive individual rewards reaching milestones (20/50/100 points)…” #4 | 1 (6%) | 4 |
| **8.1 Behavioural practice/rehearsal** | Repetition and substitution | “…The activities involved tasting different fruits and vegetables, performing various PA, and using their creativity to compose songs about healthy habits…” | 1 (6%) | 1 |
