# Supplementary material for: A Systematic Literature Review of Peer-led Strategies for Promoting Physical Activity Levels of Adolescents
Source: Health Educ Behav. 2021 Oct 11;49(1):41–53. doi: 10.1177/10901981211044988 (PMC8892039; doi:10.1177/10901981211044988)
Supplement: Supplementary material [file sj-docx-5-heb-10.1177_10901981211044988.docx]

*Summary of peer leadership training*

| **Study** | **Trained by** | **Training length** | **Training content** |
| --- | --- | --- | --- |
| Aceves-Maetins et al. (2017) | Conducted by health promotion and communication specialists. Teacher support available within sessions | Initial one-off training session: 4 hours.  Weekly sessions: 1.3 hours, 24 weeks over 2 years (12 weeks per year). | Social marketing principles and healthy lifestyle theory. Aim of the sessions were to train ACCs on health promotion, health education, communication and social media so that they could design the challenge activities for their peers. Taught about the primary and secondary objectives of the intervention, and the ACCs then had to design the challenges for their schoolmates to accomplish the defined objectives. |
| Bell et al. (2014) | Trainers were from an experienced training company specialising in health and well-being and masters students in nutrition, health and exercise also recruited. Two lead trainers, one support trainer with a teacher present | 2 day initial training away from school.  4 follow up sessions in school. | Training aimed to increase knowledge and skills, and influence behaviour through practical and interactive sessions including drama, food preparation, IT and games.  The key messages in relation to PA were to increase the volume of physical activity and the amount of MVPA and to decrease the time spent sedentary. |
| Carlin et al. (2018) | Researcher | 1x Lunchtime session | Consideration of safety concerns and emphasised the importance of the walks being performed at a brisk pace for MVPA. Leaders were taught to recognise the physiological indicators of moderate intensity. Provided with a copy of training manual. Supported in their role through an online private social media group moderated by the researcher, where they were provided with advice on facilitating the intervention. |
| Corder et al. (2016) | Mentors, teachers and peer leaders trained and provided with ongoing support by the intervention facilitators | 1 hour | No further information provided |
| Cui et al. (2012) | Research staff | x3 90 min workshops after school in the school (3 consecutive days) | Four components were explained and practiced, with the aim of enabling peer leaders to successfully deliver the lessons to their classmates. Leaders encouraged to learn skills to actively interact with peers and to facilitate interaction between peers. Before each peer education lesson, school doctors or class teachers had a meeting with peer leaders to clarify each peer leader’s responsibility |
| Foley et al. (2017) | Volunteer university students from health and education faculties who had received SALSA educator training from project staff. | 1 day workshop | Aim for leaders to successfully deliver the SALSA lessons to peers. Provided with a scripted manual to use as a guide and given the opportunity to practice delivery of the lesson content in groups in front of their peers and educators. Feedback was provided after the practice from SALSA educators to develop peer leaders’ educator skills. |
| Gobbi et al. 2017 | PE teacher and SEN teacher | 2 x 60-min sessions | Session 1: Communication strategies, teaching and feedback techniques, how to give proper physical assistance during different activities, general evidence on modelling mechanism.  Session 2- Practical work |
| Haapala et al. (2017) | Not reported | Not reported | Training not reported |
| Jenkinson et al.  (2018) | Not reported | 2 days | ‘Train the trainer’ model used. Focused on the development of leadership skills to enable leaders to lead a team of 4-6 younger students in a series of challenges. Also participated in the challenges in which the role-modelling of both leader and participants took place. |
| Lubans et al. (2008) | Not reported | Not reported | Training not reported |
| Lubans et al. (2010) | Researchers | 3 x 30 mins | Interactive seminars revised key PA and nutrition recommendations and behavioural strategies to support the student-directed implementation of the lunch-time activities |
| Lubans et al. (2011) | Researchers | 3 x 30 mins | Leadership seminars emphasized the qualities of good leaders and provided students with an opportunity to develop their leadership and instruction skills. |
| Lubans et al. (2016) | Researcher | 3 x 20 min seminars | Seminars provided key information surrounding the program's components and behavioural messages including current recommendations regarding youth physical activity, screen-time, and resistance training, and outlined the student leadership component of the intervention. |
| Owen et al. (2018) | University students as “mentors” | Half day in University campus and series of weekly sessions | Leadership and educational sessions delivered to the group of peer leaders, incorporating information on: PA; health, motivation; barriers to PA; ideas on how to increase PA; ideas on how to encourage peers to be more physically active; and social support for their role. Informed of their roles in the project and discussed with their mentors how best to fulfil their roles and responsibilities. Leaders were encouraged to disseminate the information they had learnt through their educational sessions to their friends and peers through informal discussions. |
| Sebire et al. (2018) | Two peer-supporter trainers who have either health promotion/PA knowledge and/or experience of facilitating group work with young people  Attended a 3-day (≈15 h) education programme (train the trainers). | 2 day course followed up with a 1-day top up course 5 weeks later (out of school) | Developed skills, knowledge and confidence to promote PA amongst peers. Aimed to build leaders’ perceived autonomy, competence and sense of social support for being a peer-supporter and for PA. Top up session focused on sharing experiences, problem solving and reinforcing key messages about PA and peer-supporting.  PA content- Goal setting, confidence and competence, busting barriers, PA knowledge, fitting PA in.  Peer supporter content-Identifying personal peer support attributes, identifying peer supporter skills, i.e where, when and who to give support, communication skills, listening skills, peer supporter role play. |
| Smith et al. (2014) | Researchers | 3 x 20 min | Seminars provided key information surrounding the program’s components and behavioural messages, including current recommendations regarding youth physical activity, screen-time, and resistance training, and also outlined the student leadership component. |
| Tymms et al. (2016) | Intervention teachers | 6 weekly sessions | In advance of each session leaders were trained on general principles of mentoring and preparation for weekly task. Leaders provided with weekly training sessions by the intervention teachers in advance of each mentoring session. This sought to provide them with the requisite knowledge about PA, the behavioural techniques used and the skills/confidence to act as a mentor. |
| Utter et al. (2011) | Intervention coordinator hired by research team | Weekly meetings  Length of specific training courses not provided | Received training in nutrition, activity, and health and opportunities for building leadership skills and planning intervention activities. Intervention coordinator facilitated the student health councils to ensure they had the resources and capacity to achieve their goals. Also supported by school management for any environmental changes. 100 students attended an empowerment course and 60 students attended a gym course for goal setting and gain confidence as role models |
